# Supplementary material for: The first mitotic division of human embryos is highly error prone
Source: Nat Commun. 2022 Nov 8;13:6755. doi: 10.1038/s41467-022-34294-6 (PMC9643329; doi:10.1038/s41467-022-34294-6)
Supplement: Supplementary file 1 — Supplementary Information [file 41467_2022_34294_MOESM1_ESM.pdf]

Supplementary Information for

The first mitotic division of human embryos is highly error prone

Cerys E. Currie[1,3†], Emma Ford[1,3†], Lucy Benham Whyte[2], Deborah M. Taylor[2], Bettina P. Mihalas[5], Muriel Erent[1,3], Adele L. Marston[5], Geraldine M. Hartshorne[1,2,4*] and Andrew D. McAinsh[1-4*]

[1]Division of Biomedical Sciences, Warwick Medical School, University of Warwick, Coventry CV4 7AL, UK.
[2]University Hospitals Coventry and Warwickshire NHS Trust, Coventry CV2 2DX, UK.
[3]Centre for Mechanochemical Cell Biology, University of Warwick, Coventry CV4 7AL, UK.
[4]Centre for Early Life, University of Warwick, Coventry CV4 7AL, UK.
[5]Wellcome Centre for Cell Biology, University of Edinburgh, Edinburgh, UK.

†these authors contributed equally
*Correspondence to a.d.mcainsh@warwick.ac.uk or geraldine.hartshorne@warwick.ac.uk

Supplementary Figure 1

a.

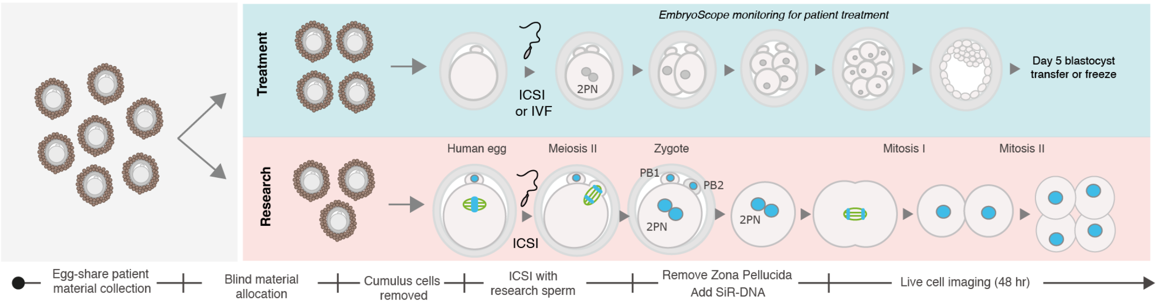

b.

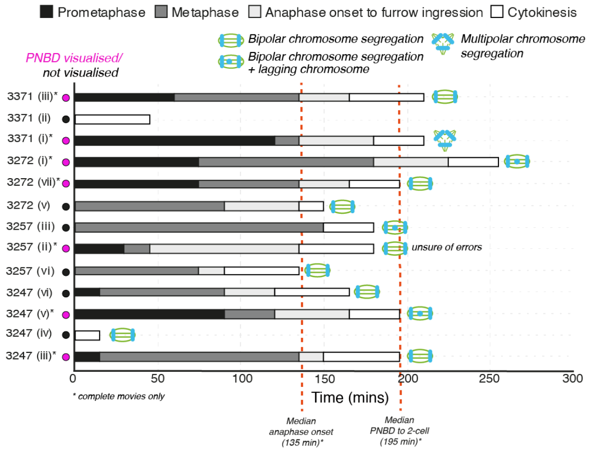

Figure S1: Egg-share to research embryos (a) Schematic outlining key steps in the egg-share-to-research protocol and embryo preparation for chromosome imaging. (b) History plots of mitotic timings of egg-share embryos undergoing chromosome imaging. Coloured bars denote timings of critical stages during the first embryonic mitosis. Pink/black dots indicate whether pronuclear break down (PNBD) was visualised during filming, and red lines indicate median times for the population of complete movies only. The research embryos of patients 3247, 3272 and 3257 were imaged using a widefield microscope, the research embryos of patients 3371 were imaged using a spinning disk microscope. Source data are provided as a Source Data file.

Supplementary Figure 2

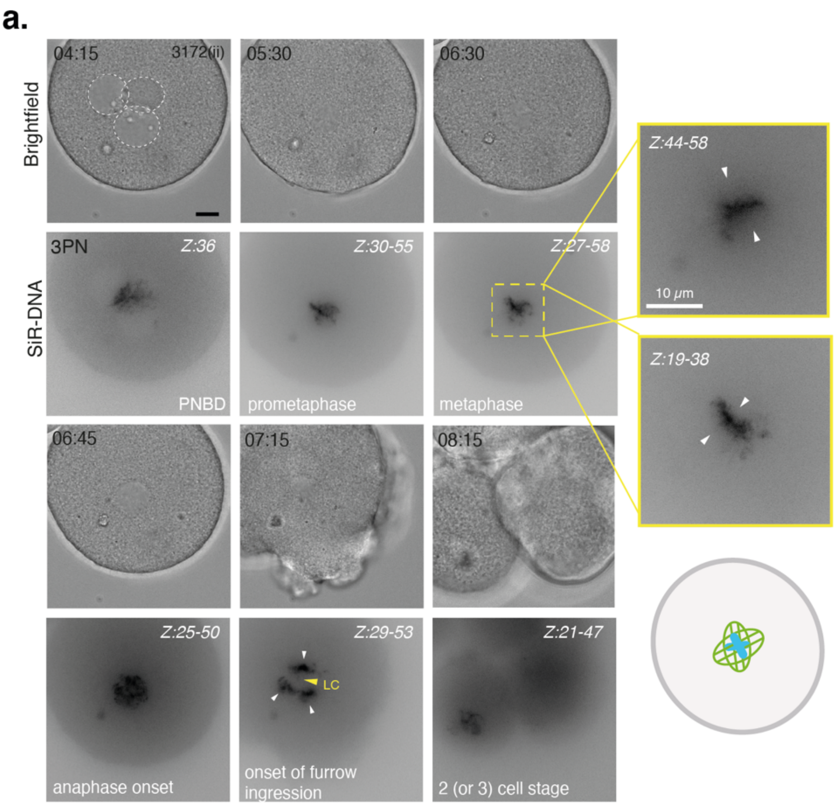

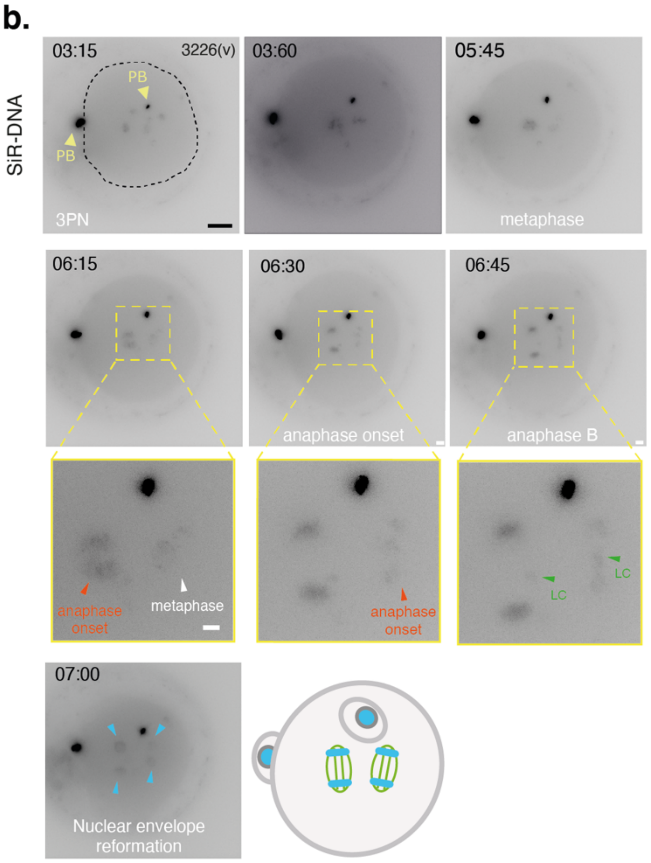

Figure S2: Evidence for dual spindles during the first embryonic mitosis in deselected human embryos. (a) Time lapse imaging of an IVF-fertilised 3PN human embryo progressing through mitosis I in the presence of an apparent perpendicular dual spindle. Chromosomes were visualised using SiR-DNA dye. Chromosome images are a maximum intensity projection of all 60 Z-slices, except zoom panels where Z is shown. Time in hours:mins, scale bar: 20 µm. Bottom right, schematic showing inferred position of the two spindles based on chromosome positions. (Embryo 3172ii). (b) Time lapse imaging of an IVF-fertilised 3PN human embryo progressing through mitosis I in the presence of a separated dual spindle. Note initiation of anaphase in left spindle before right spindle at 06:15. Green arrow heads indicate lagging chromosomes in both spindles (06:45), while blue arrow heads indicate formation of four nuclei (07:00). Chromosomes were visualised using SiR-DNA dye. Chromosome images are a maximum intensity projection of all 60 Z-slices. Time in hours:mins, scale bar: 20 µm, inset 5 μm. Bottom right, schematic showing inferred position of the two spindles (and polar bodies) based on chromosome positions. (Embryo 3226v).

Supplementary Figure 3

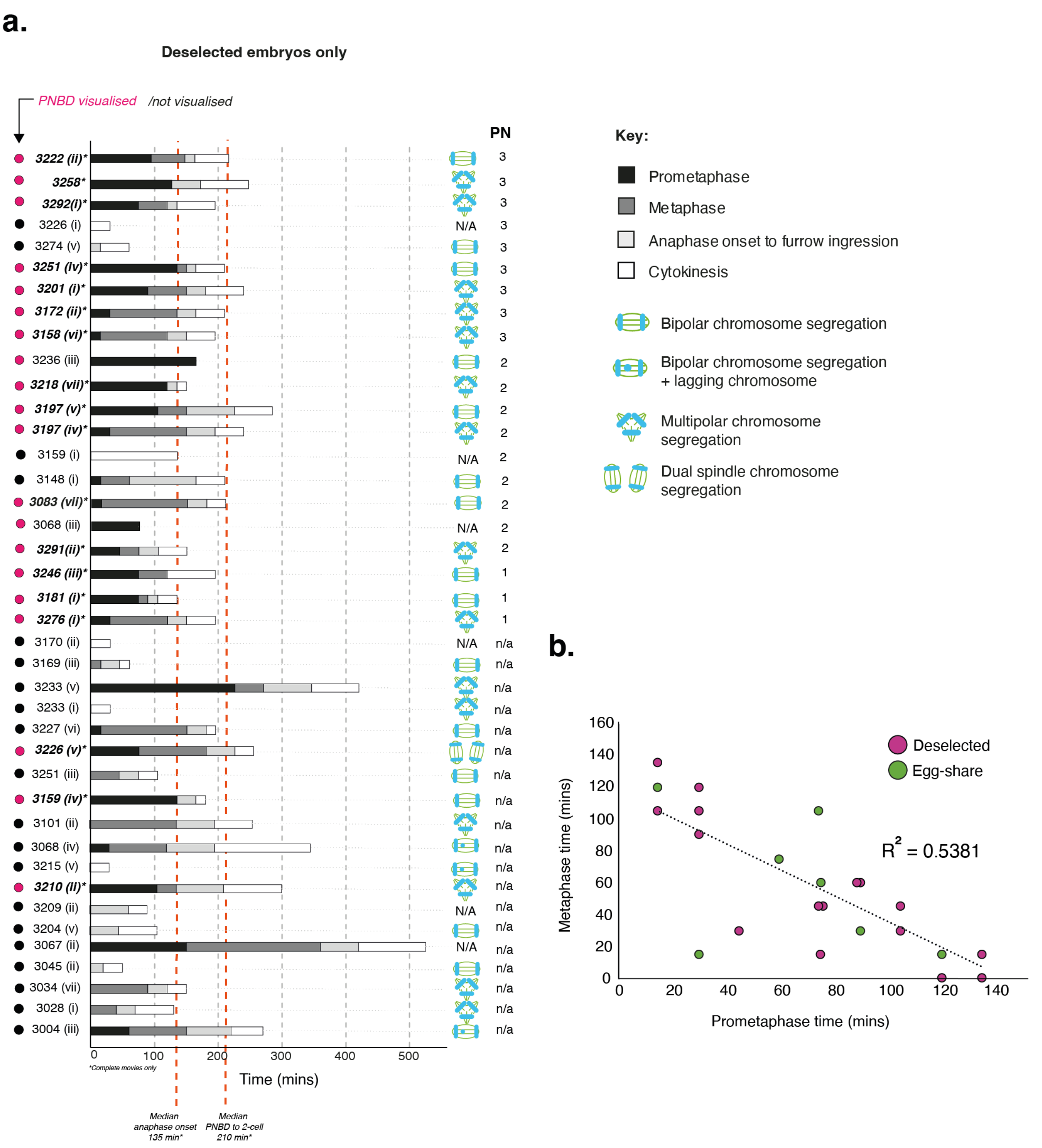

Figure S3: The first embryonic mitosis in deselected embryos. (a) History plots of deselected human embryos undergoing the first embryonic mitosis, black and grey bars denote timings of critical stages during the first embryonic mitosis and cartoons indicate phenotypes observed. Pink/black dots indicate whether pronuclear break down (PNBD) was visualised during filming. PN; pronuclei status before PNBD. Red lines indicate median durations for mitotic phases, for the population of complete movies only (marked by asterisks). (b) Timings of metaphase and prometaphase from deselected and egg-share embryos were both stages were filmed in entirety were plotted against each other. R^2 0.5881, linear fit plotted using excel. Source data are provided as a Source Data file.

Supplementary Figure 4

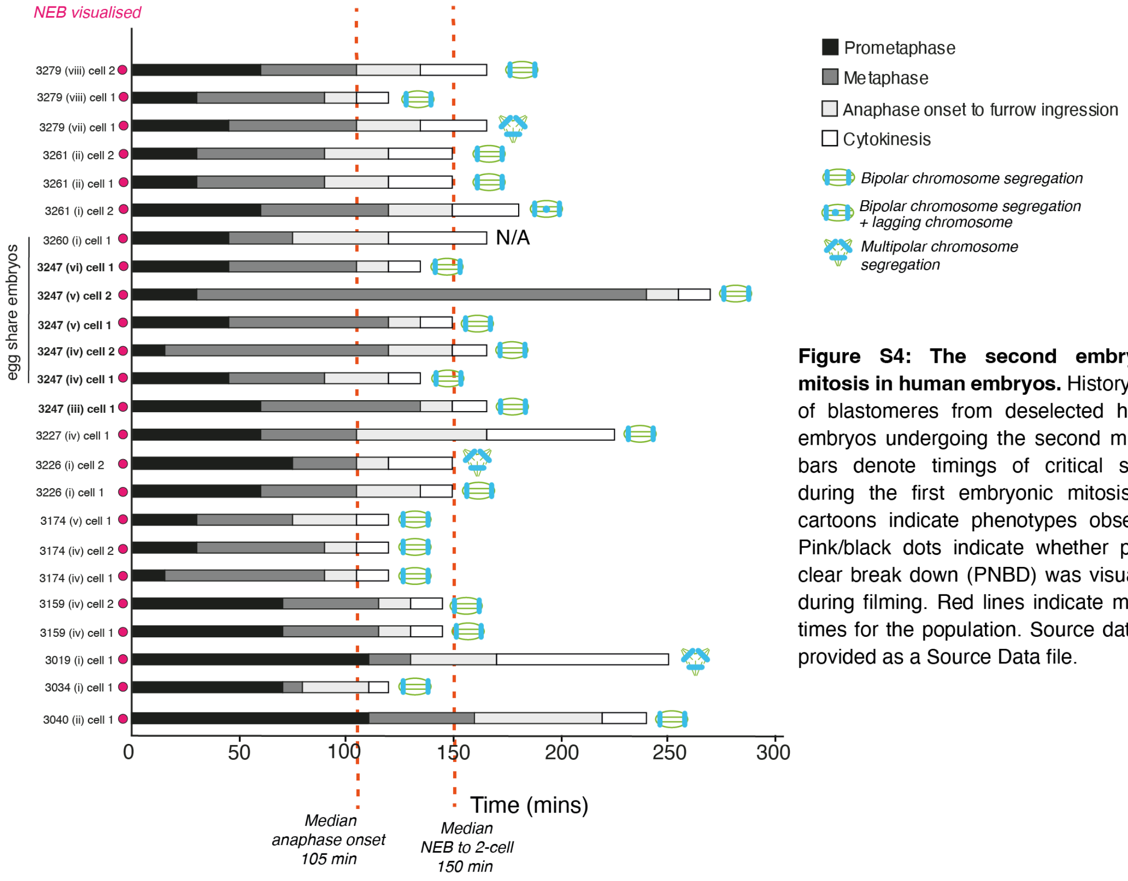

Figure S4: The second embryonic mitosis in human embryos. History plots of blastomeres from deselected human embryos undergoing the second mitosis, bars denote timings of critical stages during the first embryonic mitosis and cartoons indicate phenotypes observed. Pink/black dots indicate whether pronuclear break down (PNBD) was visualised during filming. Red lines indicate median times for the population. Source data are provided as a Source Data file.

Embryo number	Prometaphase	Metaphase	Anaphase onset to furrow ingression	Cytokinesis	PNBD to 2-cell	PNBD imaged?	PNs	Chromosome segregation	Lagging chromosomes?	Maternal age
3247 (iii) - egg share	15	120	15	45	195	YES	2PN	Bipolar	NO	29
3247 (iv) - egg share	N/A	N/A	N/A	15	15	NO	2PN	Bipolar	NO	29
3247 (v) - egg share	90	30	45	30	195	YES	2PN	Bipolar	YES	29
3247 (vi)- egg share	15	75	30	45	165	NO	2PN	Bipolar	NO	29
3257 (vi)- egg share	N/A	75	15	45	135	NO	2PN	Bipolar	NO	19
3257 (ii) - egg share	30	15	90	45	180	YES	2PN	Bipolar	N/A	19
3257 (iii) - egg share	N/A	150	0	30	180	NO	2PN	Bipolar	YES	19
3272 (v)- egg share	N/A	90	45	15	150	NO	2PN	Bipolar	NO	31
3272 (vii)- egg share	75	60	30	30	195	YES	2PN	Bipolar	NO	31
3272 (i)- egg share	75	105	45	30	255	YES	2PN	Bipolar	YES	31
3371 (i) - egg share	120	15	45	30	210	YES	2PN	Multipolar	NO	32
3371 (ii) - egg share	N/A	N/A	N/A	45	N/A	NO	2PN	N/A	N/A	32
3371 (iii) - egg share	60	75	30	45	210	YES	2PN	Bipolar	NO	32

Table S1: Mitosis I timings of human embryos from the egg sharer programe. Chromosomes were stained with SiR-DNA, embryos of patients 3247, 3257 and 3272 were imaged with a widefield microscope and the embryos of patient 3371 were imaged with a spinning disk confocal microscope.

Embryo number	Prometaphase	Metaphase	Anaphase onset to furrow ingression	Cytokinesis	PNBD to 2-cell	PNBD imaged?	PNs	Chromosome segregation	Lagging chromosomes?	Maternal age
3004 (iii)	60	90	70	50		NO		Bipolar	YES	37
3028 (i)	N/A	40	30	60		NO		Multipolar	NO	35
3034 (vii)	N/A	90	30	30		NO		Multipolar	YES	28
3045 (ii)	N/A	N/A	20	30		NO		Bipolar	NO	31
3067 (ii)	150	210	60	105		NO		NA	YES	23
3068 (iii)	75	N/A	N/A	N/A		YES	2PN	NA	N/A	29
3068 (iv)	30	90	75	150		NO		Bipolar	YES	29
3083 (vii)	15	135	30	30	210	YES	2PN	Bipolar	NO	45
3101 (ii)	N/A	135	60	60		NO		Multipolar	NO	38
3148 (i)	15	45	105	45		NO	2PN	Bipolar	NO	38
3158 (vi)	15	105	30	45	195	YES	3PN	Multipolar	NO	30
3159 (iv)	135	0	30	15	180	YES		Bipolar	NO	38
3159 (i)	N/A	0	0	135		NO	2PN	NA	N/A	38
3169 (iii)	N/A	15	30	15		NO		Bipolar	NO	35
3170 (ii)	N/A	N/A	N/A	30		NO		NA	N/A	36
3172 (ii)	30	105	30	45	210	YES	3PN	Multipolar	YES	39
3181 (i)	75	15	15	30	135	YES	1PN	Bipolar	NO	31
3197 (v)	30	120	45	45	240	YES	2PN	Multipolar	NO	30
3197 (vi)	105	45	75	60	285	YES	2PN	Bipolar	NO	30
3201 (i)	90	60	30	60	240	YES	3PN	Multipolar	NO	38
3204 (v)	N/A	N/A	45	60		NO		Bipolar	NO	30
3209 (ii)	N/A	N/A	60	30		NO		NA	N/A	30
3210 (ii)	105	30	75	90	300	YES		Multipolar	YES	37
3215 (v)	N/A	N/A	0	30		NO		Bipolar	YES	31
3218 (vii)	120	0	15	15	150	YES	2PN	Multipolar	N/A	28
3222 (ii)	90	60	15	60	225	YES	3PN	Bipolar	N/A	35
3226 (i)	N/A	N/A	N/A	30		NO		NA	NO	31
3227 (v)	75	105	45	30	255	YES	3PN	Multipolar (2 bipolar spindles)	YES	40
3227 (vi)	15	135	30	15		NO		Bipolar	NO	40
3233 (i)	N/A	N/A	N/A	30		NO		Multipolar	N/A	33
3233 (v)	225	45	75	75		NO		Multipolar	YES	33
3237 (iii)	165	N/A	N/A	N/A		YES	2PN	Bipolar	N/A	35
3246 (iii)	75	45	0	75	195	YES	1PN	Bipolar	NO	33
3251 (iii)	N/A	45	30	30		NO		Bipolar	NO	32
3251 (iv)	135	15	15	45	210	YES	3PN	Bipolar	NO	32
3274 (v)	N/A	N/A	15	45		NO	3PN	Bipolar	NO	34
3276 (i)	30	90	30	45	195	YES	1PN	Multipolar	No	37
3291(ii)	45	30	30	45	150	YES	2PN	Multipolar	YES	31
3292 (i)	75	45	15	60	195	YES	3PN	Multipolar (multiple spindles)	YES	33
3259	90	45	45	75	255	YES	3PN	Multipolar	NO	34

Table S2: Mitosis I timings of deselected human embryos. Chromosomes stained with SiR-DNA and imaged using a widefield microscope.

Name	Embryo type	Prometaphase	Metaphase	NEBD to Anaphase onset	Anaphase onset to furrow ingression	Cytokinesis time	NEB to 2 cell	Errors	NEB?
3040 (ii) cell 1	deselected	110	50	160	60	20	240	bipolar no lagging	Yes
3034 (i) cell 1	deselected	70	10	80	30	10	120	bipolar no lagging	yes
3019 (i) cell 1	deselected	110	20	130	40	80	250	**multipolar**	yes
3159 (iv) cell 1	deselected	70	45	115	15	15	145	bipolar no lagging	yes
3159 (iv) cell 2	deselected	70	45	115	15	15	145	bipolar no lagging	yes
3174 (iv) cell 1	deselected	15	75	90	15	15	120	bipolar no lagging	yes
3174 (iv) cell 2	deselected	30	60	90	15	15	120	bipolar no lagging	yes
3174 (v) cell 1	deselected	30	45	75	30	15	120	bipolar no lagging	yes
3226 (I) cell 1	deselected	60	45	105	30	15	150	bipolar no lagging	yes
3226 (I) cell 2	deselected	75	30	105	15	30	150	**Multipolar**	yes
3227 (IV) cell 1	deselected	60	45	105	60	60	225	bipolar no lagging	yes
3247 (iii) cell 1	egg sharer	60	75	135	15	15	165	bipolar no lagging	yes
3247 (iv) cell 1	egg sharer	45	45	90	30	15	135	bipolar no lagging	yes
3247 (iv) cell 2	egg sharer	15	105	120	30	15	165	bipolar no lagging	yes
3247 (v) cell 1	egg sharer	45	75	120	15	15	150	bipolar no lagging	yes
3247 (v)cell 2	egg sharer	30	210	240	15	15	270	bipolar no lagging	yes
3247 (vi) cell 1	egg sharer	45	60	105	15	15	135	bipolar no lagging	yes
3260 (i) cell 1	deselected	45	75	120	45	15	135	Bipolar - but can't see for errors	yes
3260 (i) cell 2	deselected	60	60	120	15	30	165	bipolar no lagging	yes
3260 (ii) cell 1	deselected	60	30	90	30	15	135	bipolar no lagging	yes
3260 (ii) cell 2	deselected	45	45	90	30	15	135	**Multipolar**	yes
3278 (viii) cell1	deselected	45	45	90	15	30	135	bipolar no lagging	yes
3278 (viii) cell2	deselected	60	45	105	30	45	180	bipolar no lagging	yes

Table S3: Mitosis II timings of human embryos. Chromosomes stained with SiR-DNA and imaged using a widefield microscope.
