# Supplementary material for: The first mitotic division of human embryos is highly error prone
Source: Nat Commun. 2022 Nov 8;13:6755. doi: 10.1038/s41467-022-34294-6 (PMC9643329; doi:10.1038/s41467-022-34294-6)
Supplement: Supplementary file 3 — Description of Additional Supplementary Files [file 41467_2022_34294_MOESM3_ESM.pdf]

Description of Additional Supplementary Files

File Name: Supplementary Movie 1
Description: Embryo 3247(iii) (Fig 1a) egg sharer embryo undergoing mitosis 1, chromosomes visualised using sirDNA, imaged using a widefield microscope.

File Name: Supplementary Movie 2
Description: Emryo 3371 (i) (Fig 1e) egg sharer embryo undergoing mitosis 1 with multipolar chromosome segregation, chromosomes visualised using sirDNA, imaged using a spinning disk confocal microscope.

File Name: Supplementary Movie 3
Description: Embryo 3272(i) (Fig 1e) egg sharer embryo undergoing mitosis 1 with lagging chromosome, chromosomes visualised using sirDNA, imaged using a widefield microscope.

File Name: Supplementary Movie 4
Description: Embryo 3215(v) (Fig 2d) deselected embryo undergoing mitosis 1 with micronuclei formation, chromosomes visualised using sirDNA, imaged using a widefield microscope.

File Name: Supplementary Movie 5
Description: Embryo 3172(ii) (Fig S2a), deselected embryo undergoing mitosis 1 with the appearance of two overlapping spindles, chromosomes visualised using sirDNA, imaged using a widefield microscope.

File Name: Supplementary Movie 6
Description: Embryo 3226(v) (Fig S2b), deselected embryo undergoing mitosis 1 with two separated spindles, chromosomes visualised using sirDNA, imaged using a widefield microscope.

File Name: Supplementary Movie 7
Description: Embryo 3471 (Fig 3b), deselected embryo undergoing mitosis 1 with a lagging chromosome, chromosomes visualised using H2B mCherry injection, imaged using a spinning disk confocal microscope.

File Name: Supplementary Movie 8
Description: Embryo 3457 (Fig 3c), deselected embryo undergoing mitosis 1 with a slow congressing single chromosome, plus multiple lagging chromosomes, chromosomes visualised using H2B mCherry injection, imaged using a spinning disk confocal microscope.

File Name: Supplementary Movie 9
Description: Embryo 3467_2 Fig 3d, deselected embryo undergoing mitosis 1 with multipolar chromosome segregation, chromosomes visualised using H2B mCherry injection, imaged using a spinning disk confocal microscope.

File Name: Supplementary Movie 10
Description: Clinical treatment embryo 16, mononucleated at the 2-cell stage. Filmed in the IVF clinic using Embryoscope.

File Name: Supplementary Movie 11
Description: Clinical treatment embryo 46, micronuclei (>10µm) formed at the 2-cell stage. Filmed in the IVF clinic using Embryoscope.

File Name: Supplementary Movie 12
Description: Clinical treatment embryo 53, a nuclear variant was formed at the 2-cell stage. Filmed in the IVF clinic using Embryoscope.
