# Supplementary material for: The first mitotic division of human embryos is highly error prone
Source: Nat Commun. 2022 Nov 8;13:6755. doi: 10.1038/s41467-022-34294-6 (PMC9643329; doi:10.1038/s41467-022-34294-6)
Supplement: Supplementary file 16 — Reporting Summary [file 41467_2022_34294_MOESM16_ESM.pdf]

nature portfolio

Corresponding author(s): Prof. Andrew McAinsh
Last updated by author(s): 13-09-2022

Reporting Summary

Nature Portfolio wishes to improve the reproducibility of the work that we publish. This form provides structure for consistency and transparency in reporting. For further information on Nature Portfolio policies, see our Editorial Policies and the Editorial Policy Checklist.

Statistics

For all statistical analyses, confirm that the following items are present in the figure legend, table legend, main text, or Methods section.

n/a	Confirmed	
☐	☒	The exact sample size (*n*) for each experimental group/condition, given as a discrete number and unit of measurement
☐	☒	A statement on whether measurements were taken from distinct samples or whether the same sample was measured repeatedly
☐	☒	The statistical test(s) used AND whether they are one- or two-sided<br>*Only common tests should be described solely by name; describe more complex techniques in the Methods section.*
☐	☒	A description of all covariates tested
☐	☒	A description of any assumptions or corrections, such as tests of normality and adjustment for multiple comparisons
☐	☒	A full description of the statistical parameters including central tendency (e.g. means) or other basic estimates (e.g. regression coefficient) AND variation (e.g. standard deviation) or associated estimates of uncertainty (e.g. confidence intervals)
☐	☒	For null hypothesis testing, the test statistic (e.g. *F*, *t*, *r*) with confidence intervals, effect sizes, degrees of freedom and *P* value noted<br>*Give P values as exact values whenever suitable.*
☒	☐	For Bayesian analysis, information on the choice of priors and Markov chain Monte Carlo settings
☒	☐	For hierarchical and complex designs, identification of the appropriate level for tests and full reporting of outcomes
☒	☐	Estimates of effect sizes (e.g. Cohen's *d*, Pearson's *r*), indicating how they were calculated

Our web collection on statistics for biologists contains articles on many of the points above.

Software and code

Policy information about availability of computer code

Data collection	No software was used for data collection
Data analysis	MATLAB R2020a, FIJI 2.3.0, OMERO web v5.14.0

For manuscripts utilizing custom algorithms or software that are central to the research but not yet described in published literature, software must be made available to editors and reviewers. We strongly encourage code deposition in a community repository (e.g. GitHub). See the Nature Portfolio guidelines for submitting code & software for further information.

Data

Policy information about availability of data

All manuscripts must include a data availability statement. This statement should provide the following information, where applicable:

- Accession codes, unique identifiers, or web links for publicly available datasets
- A description of any restrictions on data availability
- For clinical datasets or third party data, please ensure that the statement adheres to our policy

The mimimum dataset of representative movies is available through Zenodo, hosted on OMERO using the following link and login/password (public/public).

Human research participants

Policy information about studies involving human research participants and Sex and Gender in Research.

Reporting on sex and gender	The embryos used in this study were not tested for sex because this would have interfered with the study design for live cell imaging and may have affected the results due to additional intervention to embryos. Eggs were collected from female patients, and some were fertilised using sperm from their male partner. Other eggs were fertilised in the course of the research using sperm from a male donor. Embryos were the product of a male and a female. The total numbers of embryos used are as stated in the manuscript.
Population characteristics	All patients were attending a fertility clinic for IVF or ICSI treatment with a view to having a child or preserving their fertility. Female patients were aged ____ to ____ and were not menopausal. Age data is provided in supplementary tables. Patients would have received a range of diagnoses for either male partner, female partner, or both, while some couples would have infertility of unknown aetiology.
Recruitment	Embryos unsuitable for use in treatment: All patients attending the clinic and preparing for IVF or ICSI treatment were eligible to participate if they wished, but not all patients would have had material suitable for use as this would depend upon what happened during their treatment. All patients were approached in advance of their treatment by trained research nurses about various optional extras including research participation. Patients could opt in or out of the research according to their preference. This is not thought to have resulted in any bias of age or sex in the results.<br><br>Egg sharing programme: Patients could optionally access an information sheet in the clinic waiting room, describing the 'egg share to research' programme. Patients may also have received the same information sheet from clinicians running a parallel egg sharing programme (where patients share eggs with another patient). Patients seeking further information had appointments with a researcher, a research nurse, a counsellor and a medically qualified doctor before participating in the egg sharing programme. Patients were aged <33 for the egg sharing programme, with a view to ensuring that female age-related decline in oocytes had not begun.
Ethics oversight	The research was approved by the NHS Research Ethics Committee, and the Human Fertilisation and Embryology Authority, as stated in the methods section.

Note that full information on the approval of the study protocol must also be provided in the manuscript.

Field-specific reporting

Please select the one below that is the best fit for your research. If you are not sure, read the appropriate sections before making your selection.

☒ Life sciences ☐ Behavioural & social sciences ☐ Ecological, evolutionary & environmental sciences

For a reference copy of the document with all sections, see nature.com/documents/nr-reporting-summary-flat.pdf

Life sciences study design

All studies must disclose on these points even when the disclosure is negative.

Sample size	Human embryos are rare so sample size is limited. Nevertheless we studied 41 deselected embryos and 12 egg sharer embryos (from 1 egg share patients). We also studied a further 304 embryoscope movies from patients clinical treatment.
Data exclusions	Data was not excluded from analysis
Replication	As no experimental conditions are required, no replication was required We studied a large amount of human embryos to understand the phenotypes occurring in 'wild-type' mitosis 1. We observed similar phenotypes in embryos used for patient treatment, which acts as a control. All movies were analysed by 2 separate researchers to ensure validity.
Randomization	Data was not randomized as we are observing events in patient derived samples. No conditions are being compared.
Blinding	As above, no conditions are being compared so blinding was not required. The study is describing events across a patient derived dataset.

Reporting for specific materials, systems and methods

We require information from authors about some types of materials, experimental systems and methods used in many studies. Here, indicate whether each material, system or method listed is relevant to your study. If you are not sure if a list item applies to your research, read the appropriate section before selecting a response.

Materials & experimental systems

n/a	Involved in the study
☐	☒ Antibodies
☒	☐ Eukaryotic cell lines
☒	☐ Palaeontology and archaeology
☒	☐ Animals and other organisms
☒	☐ Clinical data
☒	☐ Dual use research of concern

Methods

n/a	Involved in the study
☒	☐ ChIP-seq
☒	☐ Flow cytometry
☒	☐ MRI-based neuroimaging

Antibodies

Antibodies used

α-tubulin (Mouse 1:200; T6074, Sigma), CENPC (guinea pig 1:200; MBL, PB030), goat anti-mouse Alexa Fluor 555 (1:500; A-21422, ThermoFisher Scientific) and goat anti-guinea pig Alexa Fluor 647 (1:500; A-21450, ThermoFisher Scientific).

Validation

α-tubulin (Mouse 1:200; T6074, Sigma) is validated by the manufacturer for ICC/IF, IP, microarray and WB and reported on the website https://www.sigmaaldrich.com/GB/en/product/sigma/t6074

CENPC (guinea pig 1:200; MBL, PB030) is validated by the manufacturer for ICC/IF, IP and western blot and reported on the website https://www.mblbio.com/bio/g/dtl/A/?pcd=PD030

goat anti-mouse Alexa Fluor 555 (1:500; A-21422, ThermoFisher Scientific): validated by the manufacturer for ICC/IF. Information found here: https://www.thermofisher.com/antibody/product/Goat-anti-Mouse-IgG-H-L-Cross-Adsorbed-Secondary-Antibody-Polyclonal/A-21422

goat anti-guinea pig Alexa Fluor 647 (1:500; A-21450, ThermoFisher Scientific): validated by the manufacturer for ICC/IF. Information found here: https://www.thermofisher.com/antibody/product/Goat-anti-Guinea-Pig-IgG-H-L-Highly-Cross-Adsorbed-Secondary-Antibody-Polyclonal/A-21450
